# Dacarbazine as a Positive Control in Melanoma Cell Lines (A375, SK‐MEL‐103, 1205Lu) and a Human Ex Vivo Skin Model

**DOI:** 10.1002/adbi.202500532

**Published:** 2026-01-20

**Authors:** Marcel Nani Leite, Natália Aparecida de Paula Rios, Juliana Santos Rosa Viegas, Maria Vitória Lopes Badra Bentley, Leandra Náira Zambelli Ramalho, Enilza Maria Espreafico, Marco Andrey Cipriani Frade

**Affiliations:** ^1^ Division of Dermatology Department of Internal Medicine Ribeirão Preto Medical School University of São Paulo Ribeirão Preto São Paulo Brazil; ^2^ School of Pharmaceutical Sciences of Ribeirão Preto University of São Paulo Ribeirão Preto São Paulo Brazil; ^3^ Department of Pathology and Legal Medicine Ribeirão Preto Medical School University of São Paulo Ribeirão Preto São Paulo Brazil; ^4^ Department of Cell and Molecular Biology School of Medicine of Ribeirão Preto University of São Paulo Ribeirão Preto São Paulo Brazil

**Keywords:** cell lines, dacarbazine, hOSEC model, melanoma, toxicity

## Abstract

One important step for evaluating and selecting a drug is toxicity studies, which are responsible for eliminating molecules that are considered promising for treating a certain disease based on their effectiveness in clinical studies, but are unsafe to go to the pharmaceutical market. We proposed an evaluation of dacarbazine as a positive control in toxicity effects in the context of macro‐ and micro effects represented by tissue and cell responses. A resazurin assay is used to evaluate cytotoxicity in cells (melanoma cells A375, Sk‐Mel‐103, and 1205Lu; immortalized skin cells HaCat and 3T3), and hematoxylin/eosin staining and TUNEL staining are used in skin explants. There is no toxicity demonstrated in the immortalized cells at the studied concentrations, whereas in the melanoma cells, A375 is the most sensitive to dacarbazine, with a high toxicity at all concentrations over 72 h (*p* < 0.05), Sk‐Mel‐103 showed toxicity effects only at 200 µg/mL, and 1205Lu showed no evidence of toxicity. Histological data showed that the entire skin structure of the explants is preserved, and no apoptotic cells are observed. Thus, we can conclude that cell lines behave differently when exposed to a drug, in this case Dacarbazine proved to be a good control for toxicity tests.

## Graphical abstract

The work seeked to demonstrate the drug Dacarbazine as a positive control for toxicity tests in immortalized skin cells (keratinocytes and fibroblasts) and in different neoplastic cell lines, through the assay with resazurin. In addition, an alternative model to replace animals in experimental studies, using skin explant culture (hOSEC ‐ human organotypic skin explant culture) which has been an interesting model for the study of the effect of drugs on various processes of skins, such as wound healing, inflammatory processes, malignant transformation, stress, aging and screening tests.

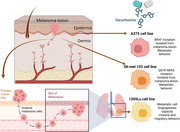

## 1 Introduction

In the development and selection of new molecules, drug candidates, vaccines, and general treatments for diseases, such as infectious and cancerous diseases, numerous tests, such as toxicity tests and carcinogenicity, mutagenicity, and teratogenicity studies, are required, all with the purpose of evaluating the potential health risks to the population [1, 2, 3].

In vitro models, such as cell and explant cultures, are important for several studies, because through these preliminary tests, the obtained results define subsequent experiments and allow selection of promising dosages [4, 5]. There is an extensive range of cytotoxicity bioassays for measuring numerous cellular functions, such as enzyme activity, ATP production, cell membrane permeability, cell adhesion, nucleotide uptake and absorption activity, which are used in several fields of biology that assist in screening drugs [6]. Among the most commonly used tests are the 3‐(4,5‐dimethylthiazolyl‐2)‐2,5‐diphenyltetrazolium bromide (MTT) assay, LDH assay, neutral red uptake (NRU) assay, trypan blue assay and Alamar blue assay (resazurin), each with their specific characteristics [6, 7, 8].

To test new drugs, it is important to use them both in cell cultures and in explants, the latter being an alternative model for decreasing the use of animals. In 1954, Charles Hume introduced the strategy of the 3Rs, that is aimed at the reduction, refinement, and replacement of animals in research laboratories [9, 10, 11]. Skin explants have been an interesting model for the study of wound healing, inflammatory processes, autoimmune diseases, malignant transformation, stress, and aging, and for screening drugs. They are easy to obtain and have great potential for dermatological research in regards to tissue and cellular responses [11]. An example of an in vitro skin model for the treatment of melanoma would be to add cancerous cells, for example, melanocytes, to this model, replacing the healthy cells, and thus perform treatment with anti‐neoplastic drugs [12]. Another model that can be used is the ex vivo model, in which a piece of the patient's tumor is removed and grown in the laboratory for testing resistant and non‐resistant drugs for the disease [13].

Human organotypic skin explant culture (hOSEC) represents an explant model that is closer to human skin under in vivo conditions, with 3D dimensions, which consists of keratinocytes, melanocytes, Langerhans cells, fibroblasts, and skin appendages such as follicles and eccrine and apocrine sweat glands [9, 11]. This model is considered efficient for testing products that need to be evaluated in terms of efficiency and safety for human skin and has been consolidated as an alternative to the use of models in experimental animals [9, 14].

Dacarbazine (DTIC) is a chemotherapy drug that was approved by the FDA in 1975. It is an alkylating agent that acts by methylating DNA bases, resulting in cell death, mainly through the apoptosis pathway. DTIC is used as a treatment for several types of cancers, such as malignant melanoma, Hodgkin's lymphoma, and pancreatic cancer [15, 16, 17]. Dacarbazine and temozolomide have been the most commonly used agents, although no study has demonstrated a significant improvement in overall survival. Despite this, these two drugs continue to represent the main therapeutic option in the second line or in subsequent lines for patients with metastatic cutaneous melanoma without actionable mutations in the BRAF gene, and they remain as third‐line or later alternatives in cases where a BRAF mutation is present [18].

DTIC has the visible characteristic of a white or slightly yellowish crystalline powder with the molecular formula C_6_H_10_N_6_O, a molecular weight of 182.18 g/mol, and a pK_a_ of 4.4; it is soluble in ethanol and acetonitrile, slightly soluble in water and anhydrous alcohol, and practically insoluble in dichloromethane. It should be protected from light and stored at 2°C–8°C to avoid degradation [19, 20].

Considering our previous results regarding DTIC absorption by hOSEC cultured for up to 24 h [9], we intend to demonstrate in this work the toxicity profile of DTIC in different normal skin cell lines, such as immortalized keratinocytes and fibroblasts, and tumoral cell lines, such as melanoma cell lines, as well as in a skin ex vivo model (hOSEC) cultured for up to 48 and 72 h. It is important to use melanoma cells and skin cells to know how the drug ages in each cell type, since the drug, as a chemotherapeutic agent, works on both malignant cells and normal cells. We seek to show the importance of DTIC as a positive control to carry out a correct selection of the in vitro / ex vivo platform for carrying out the toxicity tests for each molecule that will be tested.

## 2 Materials and Methods

### 2.1 Chemicals and Reagents

Dacarbazine ≥ 99%, hydrochloric acid (HCl), resazurin sodium salt, dimethyl sulfoxide (DMSO) and phosphate buffered saline (PBS) were obtained from Sigma–Aldrich (St. Louis, Missouri, USA), Dulbecco's modified Eagle's medium (DMEM), fetal bovine serum (FBS), antibiotics and antimycotic (AA) and trypsin were obtained from GIBCO ‐ Invitrogen Corporation (Grand Island, NY), and the water was purified with a Milli‐Q system (Millipore, Bedford, MA, USA).

### 2.2 Cell Lines

#### 2.2.1 Immortalized Cells

NIH‐3T3 (mouse fibroblast cell line) was purchased from the American Type Culture Collection (ATCC CRL‐1658, Manassas, Virginia, USA), A375 cell (human melanoma cell line) was provided by Dr. Wilson Araujo da Silva Jr. (University of São Paulo), 1205Lu cell (human melanoma cell line) was kindly provided by Dr. Meenhard Herlyn (The Wistar Institute, Philadelphia, USA), Sk‐Mel‐103 cell (human melanoma cell line) was kindly provided by Dr. Marisol Soengas (CNIO, Madrid, Spain) and HaCaT cell (human keratinocyte cell line) was acquired from the Bank of Cells of Rio de Janeiro. All cells were cultured in DMEM supplemented with 10% FBS and 1% AA and incubated at 37°C in a humidified atmosphere containing 5% CO_2_.

### 2.3 Cell Viability Assay

The viability of cells was assessed using resazurin, a dye that has a bluish tinge that is converted into the pink/purple fluorescent molecule resofurin in the presence of viable cells, demonstrating viability through mitochondrial activity.

The cells were plated in 96‐well flat‐bottomed microplates at a density of 5 × 10^3^ cells per well (200 µL volume) in triplicate and cultivated for 24 h. DTIC (2.0 mg/mL) was diluted in 50 µL of HCl (1 N) and then diluted in 1 mL of DMEM culture medium, followed by filtration through a 0.22 µm membrane. The medium was removed from the plates, and DTIC diluted in medium was added at concentrations of 10.0, 20.0, 50.0, 100.0, and 200.0 µg/mL. Culture medium without DTIC was used as the positive control (basal) for viability, and culture medium plus 20% DMSO was used as a negative control. Cell viability was evaluated at 24, 48 and 72 h. After incubation times, the culture medium was removed, and 200 µl of 0.01% resazurin solution in DMEM was added and incubated for 4 h. Then, the measurements were performed on a SpectraMax M3 microplate reader (Molecular Devices, CA, USA) using an excitation wavelength of 540 nm and an emission wavelength of 590 nm. The result was expressed as a percentage of viability, which was determined by the relationship between the optical density (OD) of the different drug concentrations divided by the OD of the positive control, multiplied by 100 [8].

### 2.4 Tissue

#### 2.4.1 Ex Vivo Model (hOSEC)

Fragments of healthy human skin originating from abdominoplasty disposal were used. After surgery, the fragments were stored in a sterile and hermetically sealed bottle and transported to the Healing and Leprosy Laboratory at Ribeirão Preto Medical School ‐ USP. The fragments were incubated with PBS plus 1.5% AA overnight at 4°C. The manipulation was performed inside a laminar flow with sterile materials, the excess subcutaneous tissue was removed, and fragments of 1 cm were sectioned with the aid of a histological punch. The fragments were placed on filter paper and a metallic grid in a 12‐well culture plate, one fragment per well, and were cultured with 2 mL of DMEM culture medium supplemented with 10% (v/v) FBS and 1% (v/v) AA [9].

As already mentioned (section “Cell viability assay”), a concentrated solution of DTIC (2.0 mg/mL) was prepared. After 24 h, the culture medium was removed from the wells, and 2 mL of test solutions containing DMEM + DTIC at a concentration of 200.0 µg/mL was added. DMEM without drug was used as the control for viability. The plates were incubated at 37°C with 5% CO_2_ for 48 and 72 h. After the incubation period, the fragments were removed and placed in 10% buffered formaldehyde solution for subsequent histological analysis.

### 2.5 Histological Study

To compare with our previously published results that assessed up to 24 h of culture [9], 3 skin fragments were removed after 48 and 72 h of culture and placed in histological cassettes containing 10% buffered formaldehyde solution for 24 h. Afterward, the samples were processed for histological analysis according to methods outlined in Andrade et al. (2017) [21]. The specimens were sectioned into 4 µm sections, placed on histological slides and stained with hematoxylin/eosin (H/E) to assess the structure of the skin. Histological images were captured using a Leica DM 4000B optical microscope equipped with a LEICA DFC 280 camera (Leica Microsystems, Germany) at 100 x and 400 x magnifications using Leica Application Suite (LAS) version 3.2.0.

### 2.6 Apoptosis Studies (TUNEL Assay)

As in the histological analysis (section “Histological study”), after processing the samples, 4 µm histological sections were made and stained with the DeadEnd Colorimetric TUNEL System kit (Promega Corporation, Madison, WI, USA) following the manufacturer's protocol for apoptosis analysis. Histological images were captured using a Leica DM 4000B optical microscope equipped with a LEICA DFC 280 camera at 200 x and 400 x magnifications using Leica Application Suite (LAS) version 3.2.0.

### 2.7 Statistical Analysis

Statistical variations on the different days were analyzed using one‐way ANOVA (variance for multiple comparisons) and are expressed as the mean value ± standard error of means (SEM). Tukey's test was used to identify statistically significant differences between the mean values determined for each group using GraphPad Prism 8 software (San Diego, CA, 176 USA). Values of *p* < 0.05 were considered significant.

## 3 Results

### 3.1 Cell Viability

**FIGURE 1:**
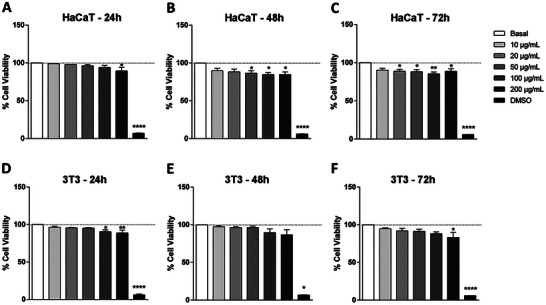
Viability of immortalized skin cells treated with DTIC. (a–c) Viability of HaCaT and (d–f) 3T3 cells at 24, 48, and 72 h. Values represent the means ± SEM (*n*=3). ^*^Corresponds to a significant difference to basal group (*p* < 0.05) by ANOVA followed by a Tukey's posttest.

**FIGURE 2:**
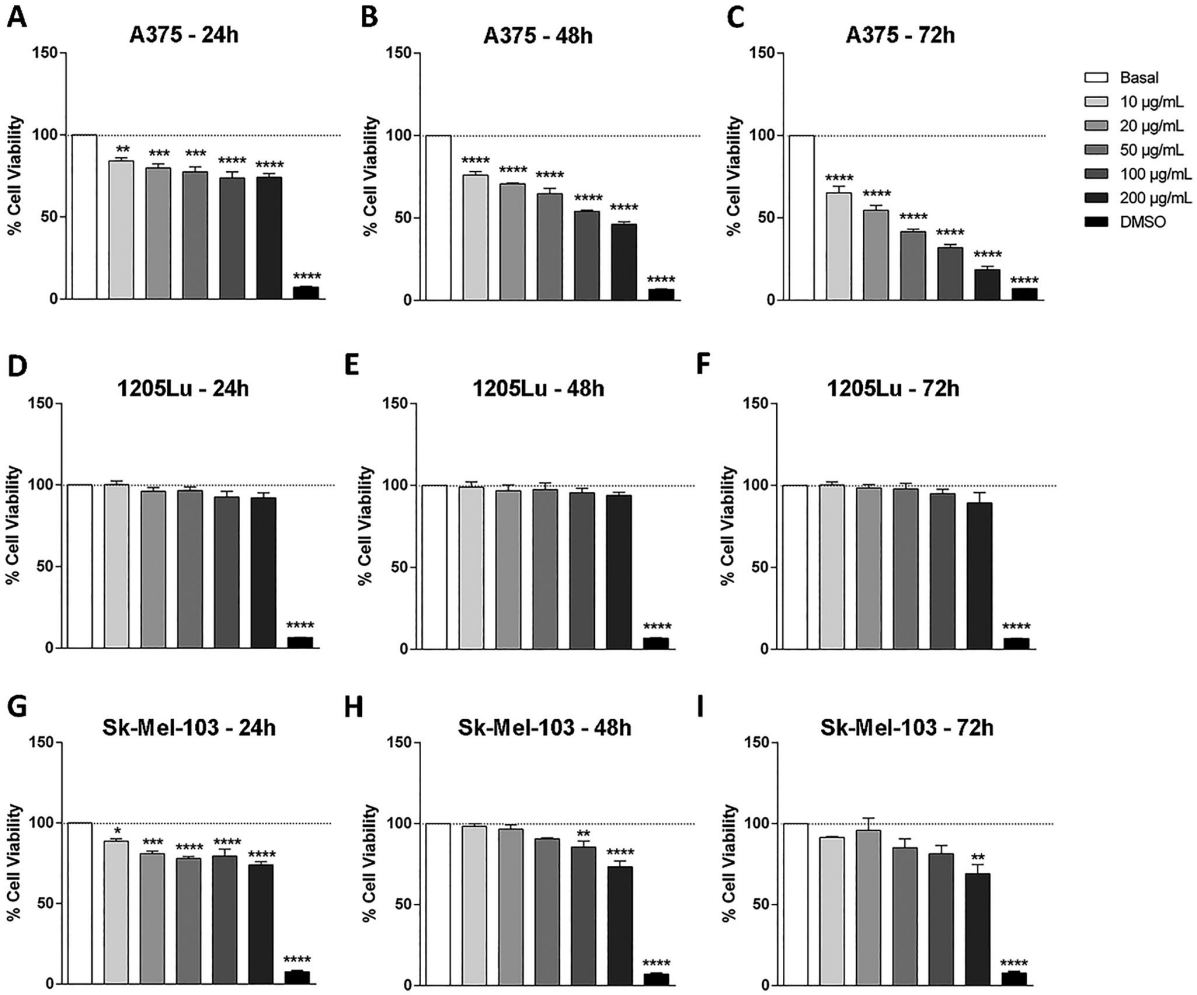
Viability of melanoma cells treated with DTIC. (a–c) Viability of A375, (d–f) 1205Lu and (g–i) Sk‐Mel‐103 cells at 24, 48, 72 h. Values represent the means ± SEM (*n* = 3). ^*^Corresponds to a significant difference to the basal group (*p* < 0.05) by ANOVA followed by a Tukey's posttest.

The immortalized skin cells HaCaT and 3T3 were evaluated first, and both presented viability above 83% at the concentrations and evaluated times of 24, 48, and 72 h (Figure 1). In the melanoma cell lines, a strong difference in viability between the lineages and time was observed. The A375 cells showed a high viability at all concentrations (above 73%) after 24 h (Figure 2a), but the viability decreased at 48 and 72 h. At 48 h, only a concentration of 10.0 µg/mL presented viability of approximately 75% (Figure 2b), and at 72 h, all concentrations were below 65% (Figure 2c). In the 1205Lu cell line, the viability was above 89% at all times and concentrations (Figure 2d–f). In the Sk‐Mel‐103 cell line, at 24 and 48 h at all concentrations, a percentage above 73% was observed; at 72 h, only at a concentration of 200.0 µg/mL was the percentage below 70% (Figure 2g–i).

### 3.2 Histological Study

**FIGURE 3:**
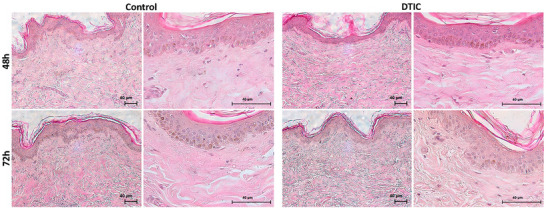
Histological analysis with H/E. Photomicrography of the skin with H/E staining, untreated (control) and treated with DTIC at 48 and 72 h (magnifications: 100 x and 400 x).

Through histological analysis with H/E staining that was used to evaluate the structure of the skin after cultivation with and without DTIC at 48 and 72 h, it was possible to observe the maintenance of the structure of the skin layers in the images. The layers of the epidermis were preserved, as well as the dermo‐epidermal junction, the papillary dermis (loose tissue) and reticular dermis (dense tissue). Nevertheless, even with DTIC treatment, no type of immune defense cell or inflammatory process was observed (Figure 3).

### 3.3 Apoptosis Analysis

**FIGURE 4:**
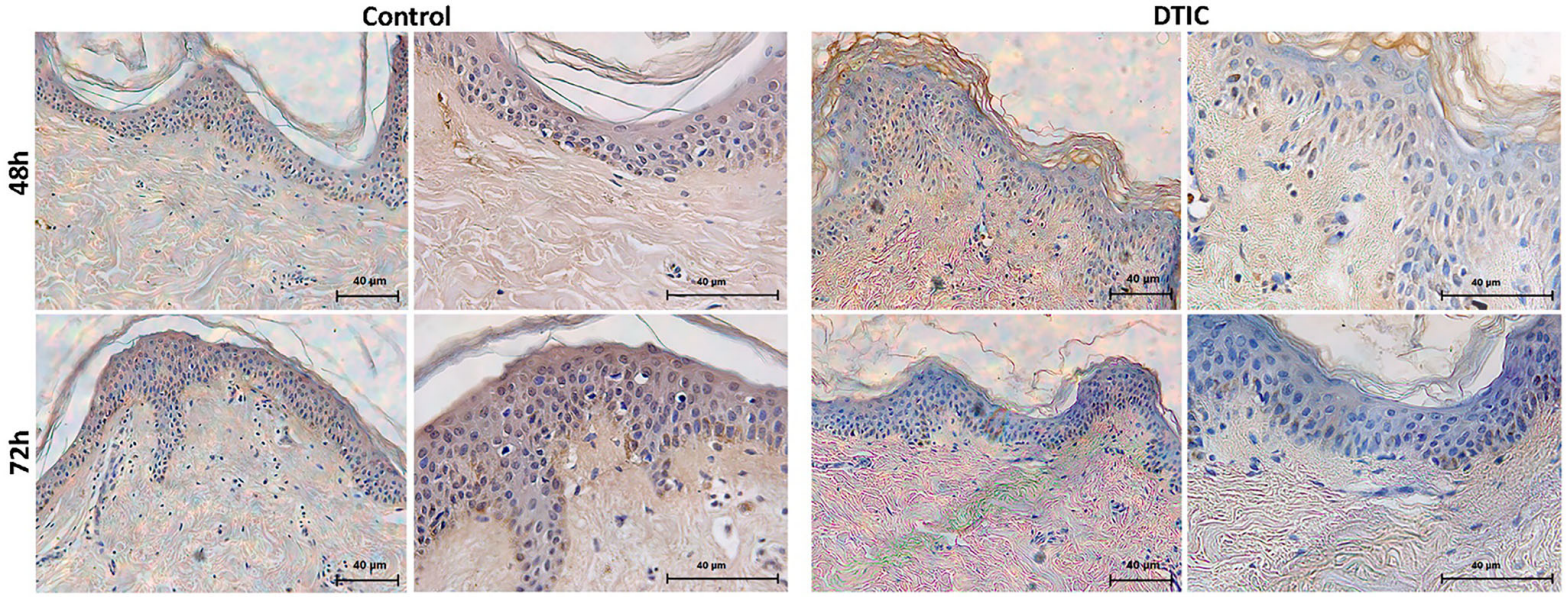
Apoptosis analysis with TUNEL. Photomicrography of the skin with TUNEL staining, untreated (control) and treated with DTIC at 48 and 72 h (magnifications: 200 x and 400 x).

In the histological analysis by TUNEL apoptosis assay, it was observed that there were no apoptotic markers in the cells in either the epidermis or dermis layers in the groups with DTIC and the control (without DTIC), thus showing that at a concentration of 200.0 µg/mL, the drug was nontoxic at 48 and 72 h (Figure 4).

## 4 Discussion

The use of in vitro methods to assess cytotoxicity is important to initiate any study of drug candidates for any disease, such as melanoma [4]. Several cell types are used in this context, which are dependent on the target organ; thus, it is important to know how to choose the right cells to test, as they may affect whether the results are relevant or give rise to equivocated interpretations. In addition to cells, explants, such skin explants, have also been an important tool to assess the safety testing of new drugs.

DTIC acts on the methylation of DNA bases, which inhibits protein synthesis and leads to cell death, mainly through apoptosis [15]. Thus, it is important to know which cell line to use to observe the effect of the drug because each cell line has its own specific characteristics, which makes it a challenge for reaching the main target.

In this work, DTIC was adopted as a well‐known drug to be used as a positive control in different cell lines as well as in an ex vivo skin model and to show the differences in its performance in toxicity tests. Chambers et al. (2021) [22] also showed in their work DTIC as a positive control compared to other drugs and other melanoma cell lines.

The immortalized skin cells HaCaT and 3T3 are already standardized cell lines widely used for drug cytotoxicity tests [23, 24, 25]. In our previous work, Leite et al. (2021) [9], we used primary cells in the same experiments used for the cells in this study, and the results of DTIC toxicity in both primary and normal immortalized cells were the same. Regarding melanoma cells, we observed that the toxicity behavior was different, which may be attributed to the sensitivity of these cells to DTIC combined with the higher metabolism that these cells present. Other works have shown the use of HaCaT and 3T3 cells as a control over other types of cancer cells to show the different drug targets [26, 27, 28, 29, 30, 31, 32].

Our results suggest that DTIC is less toxic and presents less of an effect in normal skin cells, which corroborates the idea that the concentrations tested in this work were safe. Although some normal cells are killed during treatment, it is still important to use these cells to check the amount of drug that is toxic at certain concentrations and to observe the effect of these same concentrations in cancerous cells.

We observed that unlike normal cells, there was a difference in toxicity between the melanoma cell lines. We suggest that these differences are related to the biological characteristics of these cells. The A375 cell line has two mutated genes, B‐RAF and CDKN2. These mutations are related to sun‐damaged skin. We also know that these cells are derived from a skin primary melanoma [33]. The 1205Lu lineage is highly invasive and exhibits spontaneous metastasis to the lung and liver, which makes it the appropriate cell line to evaluate metastatic events or metastatic inhibitors. However, these cells are also derived from a primary melanoma but are capable of expressing several drug resistance mechanisms [34]. Finally, Sk‐Mel‐103 and 1205Lu have characteristics related to metastatic events and drug resistance, which may be related to their resistance to RAS inhibition. This resistance confers uncontrolled growth and mutagenic events on these cells [35].

In our work, the A375 cell line proved to be the most sensitive to DTIC. At 24 h, only 200.0 µg/mL DTIC showed toxicity; however, at 48 and 72 h, practically all other concentrations were toxic. These results are in accordance with the characteristics of this lineage compared to the other two melanoma lineages. We suggest that in A375 cells, DTIC causes protein synthesis inhibition, which consequently leads to cell death. Piotrowska et al. (2019) [36] used 10.0 µm DTIC and showed that it decreases A375 proliferation by 50%. Additionally, Yang et al. (2016) [37] used concentrations similar to those in our study and showed a high cellular toxicity.

As expected, the 1205Lu and Sk‐Mel‐103 cell lines showed a high resistance at the concentrations used; toxicity effects were observed only at a concentration of 200.0 µg/mL at all times. Ralph et al. (2016) [38] used 5.0 µm DTIC as a positive control in Sk‐Mel‐103 cells at 24, 48, and 72 h and showed low toxicity, corroborating our results; on the other hand, when 5.0 µm biflorin was used, it showed high toxicity. Weber et al. (2009) [39] used 300.0 µg/mL DTIC in the 1205Lu cell line and showed that less than 20% of the cells died, also corroborating our findings, but when adding 1.0 µm ABT‐373 + 300.0 µg/mL DTIC, a percentage of death of approximately 70% was obtained after 48 h. This shows how drugs use multiple pathways to kill cells.

An alternative model for assessing toxicity is the skin explant, a complete model with cells and characteristics closer to physiological reality. H/E histology showed that the tissue was intact, with no recruitment of inflammatory cells, and that the dermis‐epidermis junction was preserved at 48 and 72 h. We also used histology with TUNEL, which marks apoptotic cells, and showed that there were no observable cellular markers for apoptosis, with viable tissue in both the epidermis and the dermis at 48 and 72 h. Frade et al. (2015) [40] showed tissue viability with antibody markers for a time up to 75 days without treatment. Leite et al. (2021) [9] showed tissue viability by a biochemical method (TTC) up to 96 h and by TUNEL and H/E histology up to 24 h.

Just like any model, the hOSEC also has some limitations, such as the absence of circulatory and nervous systems, the loss of certain cell types over time (melanocytes, Langerhans cells, and endothelial cells), as well as donor variability and absorption by the dermis [41]. Therefore, other models can be used together to obtain more robust results. Some examples include: computational models, which are used to help understand the basic biology and physiology of the human body [42]; the Franz diffusion cell, a widely used and gold‐standard model for evaluating the permeation of drugs and substances [43]; 3D cell cultures, which are an excellent alternative to animal use and exhibit characteristics that more closely resemble the complex conditions found in vivo [44]; multiorgan chips, which consist of different organotypic models that can be integrated, providing communication through fluidic systems, among others [41].

## 5 Conclusion

From these results, we can conclude that DTIC was used as a good positive control when exposed to different cell lines, whether normal or melanoma. We also demonstrated that cells behave differently when exposed to DTIC, some more sensitive to the drug and others less so, making it important to know the characteristics and action of the target drug to be used in the experiment. We also saw the importance of using normal skin cells, which shows how toxic the drug may or may not be. In addition, the use of alternative methods to avoid the use of animals is an important preclinical tool for obtaining other essential data (Supporting Information).

## Author Contributions

M.N.L. and M.A.C.F. conceived the study, curated the data, performed the formal analysis, and managed the project; M.A.C.F. secured funding and supervised, validated, and contributed to visualization; M.N.L., N.A.P., and M.A.C.F. conducted the investigation, while M.N.L., N.A.P., J.S.R.V., M.V.L.B.B., L.N.Z.R., and E.M.E. contributed to the methodology; L.N.Z.R. and E.M.E. provided resources; M.N.L., N.A.P., J.S.R.V., and M.A.C.F. drafted the manuscript, with all authors contributing to review and editing and approving the final version.

## Funding

This work was supported by Conselho Nacional de Desenvolvimento Científico e Tecnológico (CNPq) [Grant No. #423635/2018‐2], Coordenação de Aperfeiçoamento de Pessoal de Nível Superior (CAPES) [Grant No. #88882.180013/2018‐01], Fundação de Amparo à Pesquisa do Estado de São Paulo (FAPESP) [Grant No. #2016/16437‐7] and Fundação de Apoio ao Ensino, Pesquisa e Assistência do Hospital das Clínicas da Faculdade de Medicina de Ribeirão Preto‐USP (FAEPA) [Grant No. 120/2020].

## Ethical Statement

All procedures regarding the procurement of skin samples were approved by the Research Ethics Committee Ribeirão Preto Medical – process 12175/2017, with the written consent of all patients.

## Conflicts of Interest

The authors declare no conflicts of interest.

## Supporting information




**Supporting File**: adbi70084‐sup‐0001‐Data.zip.

## Data Availability

Data sharing not applicable to this article as no datasets were generated or analysed during the current study.
